# Combined Histidine and Proline Supplementation (HISPRO) Enhances Oxidative and Mitochondrial Function in Skeletal Muscle Through SIRT1-Associated Signaling

**DOI:** 10.3390/cells15100887

**Published:** 2026-05-13

**Authors:** Dohyun Lee, Jongsu Jeon, Gyuwon Huh, Seoyeong Baek, Daehun Kim, Hyeon-Ji Kang, Hoyul Lee, In-Kyu Lee, Jae-Han Jeon, Hoe-Yune Jung

**Affiliations:** 1 R&D Center, NovMetaPharma Co., Ltd., Pohang 37668, Republic of Koreajongsu@novmeta.com (J.J.); sybaek@novmeta.com (S.B.); dh.kim@novmeta.com (D.K.); 2 Division of Interdisciplinary Bioscience and Bioengineering, Pohang University of Science and Technology (POSTECH), Pohang 37673, Republic of Korea; 3 Division of Endocrinology and Metabolism, Department of Internal Medicine, Kyungpook National University Hospital, Daegu 41944, Republic of Korea; hyun13829@hanmail.net; 4 JD Bioscience Inc., Gwangju 61005, Republic of Korea; 5 Research Institute of Aging and Metabolism, Kyungpook National University, Daegu 41404, Republic of Korea; 6 Department of Internal Medicine, School of Medicine, Kyungpook National University, Kyungpook National University Hospital, Daegu 41404, Republic of Korea

**Keywords:** HISPRO, histidine, proline, oxidative metabolism, mitochondrial biogenesis, SIRT1, AMPK, skeletal muscle, muscle atrophy

## Abstract

Amino acids are key regulators of metabolism, their coordinated effects on skeletal muscle signaling and metabolic remodeling under physiological conditions remain incompletely understood. Here, we investigated whether 14 weeks of combined histidine and proline supplementation (HISPRO; 700 mg/kg) enhances skeletal muscle function through metabolic reprogramming in normal ICR mice. HISPRO significantly improved muscle performance compared with the control group, including grip strength, rota-rod, and treadmill. Histological and biochemical analyses revealed a shift toward oxidative muscle phenotype compared with the control group, with larger muscle fibers and succinate dehydrogenase-positive fibers. Consistently, HISPRO promoted mitochondrial biogenesis and oxidative metabolism compared with the control group, as evidenced by upregulation of mitochondrial regulatory genes, mitochondrial DNA copy number, citrate synthase activity, and oxidative phosphorylation (OXPHOS) complex levels in skeletal muscles. Mechanistically, HISPRO was associated with activation of the SIRT1-PGC1α-AMPK signaling axis compared with the control group, as evidenced by increased *Nampt* and *Nmnat1* expression, an elevated NAD^+^/NADH ratio, and enhanced AMPK phosphorylation. SIRT1 inhibition markedly attenuated HISPRO-induced increases in mitochondrial biogenesis markers but did not fully suppress OXPHOS protein expression, suggesting the involvement of both SIRT1-dependent and -independent mechanisms. Notably, HISPRO also improved muscle function in dexamethasone-induced muscle atrophy model. It restored mitochondrial biogenesis and function, and suppressed atrophy-related markers compared with the dexamethasone-treated group. HISPRO may contribute to improving muscle quality through coordinated metabolic regulation and could represent a complementary nutritional for supporting muscle metabolic health.

## 1. Introduction

Skeletal muscle function is a critical determinant of overall systemic health and quality of life [1]. Its decline is closely associated with aging, various diseases, and catabolic muscle-wasting conditions [1,2]. While muscle mass has traditionally been considered a primary factor underlying muscle strength, emerging evidence emphasizes that muscle quality, defined by intrinsic metabolic and contractile efficiency, is a more relevant predictor of functional capacity [3,4]. In particular, mitochondrial function plays a central role in maintaining muscle quality by regulating oxidative metabolism and cellular energy production [5,6]. Impairments in mitochondrial biogenesis and oxidative phosphorylation are key features of muscle dysfunction across diverse physiological and pathological conditions including sarcopenia and muscle atrophy [6].

Essential amino acid availability is a major regulator of skeletal muscle metabolism, and these amino acids have emerged not only as building blocks for protein synthesis but also as signaling molecules that modulate metabolic pathways [7,8]. Amino acid supplementation, including branched-chain amino acids (BCAA) and some bioactive dipeptides, has been shown to enhance muscle function [9,10]. However, most studies have focused on individual amino acids, and whether defined combinations of amino acids can cooperatively reprogram cellular metabolism to enhance muscle function remains incompletely understood.

Histidine and proline are metabolically distinct amino acids with the potential to influence complementary aspects of exercise-related muscle metabolism. Histidine contributes to the synthesis of histidine-containing dipeptides, such as carnosine, which are involved in intramuscular pH buffering, redox homeostasis, and fatigue resistance during exercise [11]. Proline supports mitochondrial redox regulation through the proline–P5C (1-pyrroline-5-carboxylate) cycle, which may contribute to metabolic adaptation under increased energy demand [12]. However, it remains unclear whether combined free histidine and proline supplementation can coordinately enhance skeletal muscle performance, oxidative remodeling, and mitochondrial function under physiological conditions, or whether such effects extend to catabolic muscle wasting. Given these distinct but potentially converging roles, we hypothesized that combined histidine and proline supplementation (HISPRO) cooperatively activates coordinated metabolic changes to enhance muscle mitochondrial function under both physiological and catabolic conditions. To test this hypothesis, we examined the effects of HISPRO using two experimental models: normal ICR mice and a dexamethasone-induced muscle atrophy model using C57BL/6 mice.

## 2. Materials and Methods

### 2.1. Animals

7-week-old male ICR and C57BL/6 mice were purchased from KOATECH (Pyeongtaek, Republic of Korea). Mice were housed in individual cages at a temperature of 23 ± 3 °C with a 12 h light/dark cycle. Mice were free to access distilled water and laboratory chow diet (Purina, St. Louis, MO, USA) ad libitum. Animal experiments were initiated after a week of adaptation. 

For the physiological condition study, a total of 24 ICR mice were randomly divided into four groups based on average body weight (CTRL, *n* = 6; Histidine 350 mg/kg, *n* = 6; Proline 350 mg/kg, *n* = 6; HISPRO 700 mg/kg, *n* = 6). Mice were orally administered 200 μL of single amino acid or combined HISPRO once daily for 14 weeks. The control group received an equal volume of vehicle (distilled water).

For the dexamethasone-induced muscle atrophy model study, A total of 22 C57BL/6 mice were randomly divided into three groups based on average body weight (CTRL, *n* = 6; Dexa, *n* = 8; Dexa + HISPRO, *n* = 8). To induce muscle atrophy, the Dexa and Dexa + HISPRO groups received dexamethasone (25 mg/kg, *i.p.*) daily for 14 days. The control group received normal saline (*i.p.*). Concomitantly, the CTRL and Dexa groups were orally administered vehicle (distilled water), whereas the Dexa + HISPRO group was orally administered HISPRO (700 mg/kg) once daily for 14 days.

### 2.2. Histidine, Proline, and HISPRO Administration

L-histidine monohydrochloride monohydrate (H5659, Sigma-Aldrich, St. Louis, MO, USA) and L-proline (P5607, Sigma-Aldrich, St. Louis, MO, USA) were dissolved in distilled water. The dose of HISPRO was selected as the minimal effective dose based on preliminary dose-finding experiments using several different doses. For single treatments, each amino acid was administered at 350 mg/kg/day. For the combined HISPRO treatment, mice received both amino acids at 350 mg/kg/day each, resulting in a total HISPRO dose of 700 mg/kg/day. The dosing solutions were freshly prepared once per week, with the amount of each amino acid adjusted according to the weekly body weight measurements. Mice were orally administered 200 μL once daily during the light phase until the end of the study. The control group received an equal volume of distilled water.

### 2.3. Grip Strength Test

Four-paw grip strength was measured using a grip strength meter (BIO-GS4, Bioseb, Vitrolles, France). In the physiological condition study, grip strength was assessed at week 9, whereas in the dexamethasone-induced muscle atrophy model, it was assessed on day 10. Mice were allowed to grasp a metal grid with all four paws and were gently pulled backward until they released the grid. The maximum force was recorded for each trial and the average grip strength (g) was calculated from three trials.

### 2.4. Rota-Rod Test

Motor coordination and balance were measured using an accelerating rota-rod (Panlab, Harvard Apparatus, Barcelona, Spain). The rota-rod test was performed after the grip strength test at week 10 in the physiological condition study and on day 11 in the dexamethasone-induced muscle atrophy model. Mice were placed on a rotating rod and the latency to fall was recorded. After a short training session, each mouse was given three test trials with accelerating rotation from 4 to 40 rpm over 300 s. The latency to fall from each trial was recorded and the mean value was calculated.

### 2.5. Treadmill

Endurance capacity was assessed at week 11 in the physiological condition study by measuring running distance using a treadmill apparatus (BLI-TR01M, BeonLi Scientific Instruments, Daejeon, Republic of Korea), as previously described with minor modifications [13]. The shock grid delivered a 0.1 mA shock, which was uncomfortable but did not physically harm the animals. Mice were habituated to the treadmill one day before the experiment. During the habituation session, mice ran on the treadmill at a speed of 15 m/min with a 5° incline for 10 min. On the following day, the endurance test was performed during the daytime, and all mice were tested on the same day. Mice performed the test according to the following protocol: 15 m/min at a 5° incline for 20 min, 17 m/min at a 5° incline for 20 min, 19 m/min at a 5° incline for 20 min, 21 m/min at a 5° incline for 15 min, 22 m/min at a 10° incline for 15 min, followed by 24 m/min at a 10° incline until exhaustion. Exhaustion was defined as the inability to resume running for 10 s despite receiving continuous shocks. Exhaustion time was recorded for each speed interval, and total running distance was calculated based on the recorded running time and treadmill speed.

### 2.6. In Vivo Oxygen Consumption Rate (VO_2_) Measurement

In the physiological condition study, whole-body oxygen consumption (VO_2_) was assessed at week 12 by indirect calorimetry using a TSE LabMaster system (TSE Systems, Bad Homburg, Germany), as described previously [14], with minor modifications. Mice were housed individually in metabolic cages under a 12 h light/12 h dark cycle at 22 ± 2 °C with free access to food and water. After a 24 h acclimation period in the calorimetry cages, VO_2_ was continuously recorded for an additional 24 h. For the present study, lights were turned on at 06:30 and off at 18:30, and VO_2_ data were averaged separately for the light (day, 06:30–18:30) and dark (night, 18:30–06:30) periods. Oxygen consumption is expressed as ml O_2_/kg/h.

### 2.7. Tissue Collection

Hindlimb muscles were collected from mice under isoflurane anesthesia. The quadriceps, gastrocnemius, soleus, tibialis anterior, and extensor digitorum longus (EDL) muscles were dissected, weighed, and stored at −80 °C until use. The gastrocnemius muscle from one leg was fixed in 10% neutral buffered formalin (HT-501128, Sigma-Aldrich, St. Louis, MO, USA) for hematoxylin and eosin (H&E) staining. A piece of gastrocnemius muscle was directly embedded in OCT compound (#4583, Sakura Finetek, Torrance, CA, USA) and snap-frozen in isopentane cooled with liquid nitrogen for succinate dehydrogenase (SDH) staining.

### 2.8. Histology

For H&E staining, the formalin fixed gastrocnemius muscles were embedded in paraffin. Thin sections were deparaffinized, rehydrated, and stained with H&E method. The stained sections were imaged using a microscope (BX53 upright microscope, Olympus, Tokyo, Japan). A total of 100–140 muscle fibers from three images were analyzed per mouse, and cross-sectional area (CSA) of individual muscle fibers was measured using ImageJ software v1.53k (National Institutes of Health, Bethesda, MD, USA) as previously described [15]. For SDH staining, as previously described [16], cryosections (10 μm thickness) of OCT-embedded samples were stained with a pre-warmed SDH staining solution (1 mg/mL nitroblue tetrazolium (NBT, N6876, Sigma-Aldrich, St. Louis, MO, USA), 27 mg/mL sodium succinate (S2378, Sigma-Aldrich, St. Louis, MO, USA) in 25 mM phosphate (S9638, Sigma-Aldrich, St. Louis, MO, USA) buffer for 30 min at 37 °C. Slides were washed three times with distilled water and mounted. The stained sections were imaged using the same microscope, and SDH-positive fibers were quantified using ImageJ software.

### 2.9. RNA Analysis

Total RNA was extracted from the muscle tissues using NucleoZOL reagent (740404.200, Macherey-Nagel, Düren, Germany). Total RNA in the amount of 1 μg of was used for cDNA synthesis using ReverTra Ace qPCR RT Master Mix (Toyobo, Osaka, Japan). Real-time qPCR (RT-qPCR) was performed using SYBR Green Realtime PCR Master mix (Toyobo, Osaka, Japan) and primer sets listed in Supplementary Table S1.

### 2.10. mtDNA Copy Number

Mitochondrial DNA content was measured by previously described D-loop quantification method [17]. Briefly, total genomic DNA was extracted from the gastrocnemius muscle using Solg™ Genomic DNA Prep Kit (SolGent, Daejeon, Republic of Korea) in accordance with the manufacturer’s instructions. To determine the mitochondrial DNA (mtDNA) copy number, quantitative real-time PCR (qPCR) was performed using the extracted total DNA as a template. The D-loop region of the mitochondrial genome was targeted for amplification to represent mtDNA levels. qPCR reactions were conducted using THUNDERBIRD^®^ Next SYBR™ qPCR Mix (Toyobo, Osaka, Japan). For normalization of the data, the 18S rRNA gene, encoded by nuclear DNA (nDNA), was utilized as an internal control. The specific primer sequences used in this study are listed in Supplementary Table S1.

### 2.11. Citrate Synthase Activity

Citrate synthase activity was measured using a commercial assay kit (CS0720, Sigma-Aldrich, St. Louis, MO, USA) according to the manufacturer’s protocol. Briefly, protein lysates of gastrocnemius muscle tissue were incubated with 0.3 mM acetyl-CoA and 0.1 mM 5,5′-dithiobis (2-nitrobenzoic acid) (DTNB), in an assay buffer at 37 °C for 1.5 min. After measuring the baseline at 412 nm, the reaction was initiated by adding oxaloacetate to a final concentration of 0.5 mM. The increase in absorbance was then measured kinetically at 37 °C for 1.5 min. Citrate synthase activity was calculated as the manufacturer’s instructions.

### 2.12. Western Blotting

Frozen gastrocnemius tissues were lysed using RIPA buffer supplemented with Halt™ Protease and Phosphatase Inhibitor Cocktail (78446, Thermo Fisher Scientific, Waltham, MA, USA). Protein lysates were separated on Bolt 4–12% Bis-Tris Plus gels (Thermo Fisher Scientific, Waltham, MA, USA) and transferred onto nitrocellulose membranes (Bio-Rad, Hercules, CA, USA). After blocking with 5% skim milk for 1 h, the membranes were incubated with primary antibodies overnight at 4 °C. The following primary antibodies were used: OXPHOS (ab110413, Abcam, Cambridge, UK), Vinculin (#13901, CST, Danvers, MA, USA), PGC-1α (NBP1-04676, Novus Biologicals, Centennial, CO, USA), p-AMPK (#2535, CST, Danvers, MA, USA), AMPK (#5831, CST, Danvers, MA, USA), GAPDH (#2118, CST, Danvers, MA, USA), Atrogin-1 (sc-166806, SCBT, Dallas, TX, USA). The membranes were washed three times with TBST and incubated with anti-rabbit or anti-mouse HRP-conjugated secondary antibodies at room temperature for 1 h. After three additional washes, signals were detected using Alliance 4.7 imaging system (UVITECH, Cambridge, UK) with ECL solution.

### 2.13. Immunoprecipitation

Protein lysates in the amount of 200 μg of were immunoprecipitated using 1 μg of antibody (α-FOXO1 (#2880, CST, Danvers, MA, USA) or α-LKB1 (sc-32245, SCBT, Dallas, TX, USA)) with 20 μL of protein G agarose beads (PROTGA-RO, Sigma-Aldrich, St. Louis, MO, USA) at 4 °C overnight. Immunocomplex-beads were then washed three times, boiled in sample buffer, and subjected to immunoblotting using anti-acetylated lysine (#9814, #9681, CST, Danvers, MA, USA), anti-FOXO1, and anti-LKB1 antibodies.

### 2.14. Cell Culture and Treatment

C2C12 myoblasts were obtained from ATCC (Manassas, VA, USA) and maintained in Dulbecco’s Modified Eagle Medium (DMEM) supplemented with 10% fetal bovine serum (FBS) and 1% antibiotic–antimycotic (15240062, Gibco, Thermo Fisher Scientific, Waltham, MA, USA). To induce differentiation into myotubes, the growth medium was replaced with differentiation medium containing DMEM supplemented with 2% horse serum. The differentiation medium was refreshed every two days for a total of three exchanges (6 days). Following differentiation, the cells were incubated in serum-free media and treated with 5 mM HISPRO (2.5 mM L-histidine (H6034, Sigma-Aldrich, St. Louis, MO, USA) + 2.5 mM L-proline (P5607, Sigma-Aldrich, St. Louis, MO, USA)), with or without 10 μM EX-527 (E7034, Sigma-Aldrich, St. Louis, MO, USA), for 24 h. The concentration of HISPRO used in C2C12 myotubes was selected based on preliminary dose-finding experiments to ensure a clear biological response without cytotoxicity.

### 2.15. Mitochondrial Density Measure

C2C12 myotubes grown on confocal culture dishes were treated for 24 h and subsequently incubated with 50 nM MitoTracker (M22426, Invitrogen, Thermo Fisher Scientific, Waltham, MA, USA) and 2 μg/mL Hoechst 33342 (B2261, Sigma-Aldrich, St. Louis, MO, USA) in serum-free media for 30 min in the 37 °C CO_2_ incubator. Cells were then washed HBSS twice and fixed with 4% PFA at room temperature for 30 min, followed by confocal imaging (Fluoview FV3000 confocal microscopy, Olympus, Tokyo, Japan). MitoTracker fluorescence was detected at wavelengths of Ex644/Em665 nm, and Hoechst 33342 at Ex350/Em461 nm. Mitochondrial density was calculated as the ratio of deep red fluorescent area to myotube area within each field using ImageJ software.

### 2.16. Statistics

Statistical analyses were performed using GraphPad Prism v.6.0. Data are presented as box-and-whisker plots (median, IQR, and min-to-max whiskers). Grubb’s test was applied a priori, and at most one outlier per group was excluded when *p* < 0.05. Differences between two groups were assessed using unpaired *t*-test, while multiple group comparisons were evaluated by one-way analysis of variance (ANOVA) followed by Sidak’s post hoc test. A *p*-value of less than 0.05 was considered statistically significant.

## 3. Results

### 3.1. Combined Histidine and Proline Supplementation (HISPRO) Enhanced Skeletal Muscle Quality and Promoted an Oxidative Muscle Phenotype

**Figure 1:**
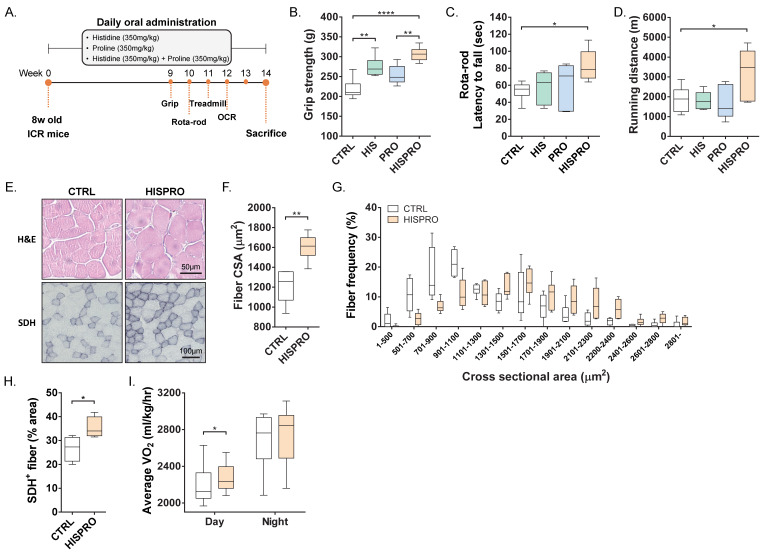
Histidine and proline (HISRPO) enhance muscle function and promote oxidative muscle phenotype. (**A**) Experimental scheme. (**B**) Grip strength. (**C**) Endurance on rota-rod test. (**D**) Total running distance on treadmill. One-way ANOVA with Sidak’s multiple comparison test. (**E**) Representative H&E-stained histological muscle fiber images of gastrocnemius. Scale bar, 25 μm (H&E) and 50 μm (SDH). (**F**) Average of muscle fiber cross-sectional area (CSA). (**G**) Distribution of muscle fiber CSA. (**H**) Population of SDH-positive muscle fibers. (**I**) In vivo oxygen consumption rate (VO_2_). Unpaired Student’s *t*-tests. *n* = 6 per group. * *p* < 0.05, ** *p* < 0.01, **** *p* < 0.0001.

We first assessed the effects of HISPRO under physiological conditions using normal ICR male mice that received oral supplementation with histidine, proline, or their combination (Figure 1A). Grip strength test showed that histidine and HISPRO significantly enhanced grip force compared with the control group (Figure 1B). The following rota-rod and treadmill tests demonstrated that only HISPRO significantly improved motor performance and endurance compared with the control group (Figure 1C,D). Although muscle mass was not significantly increased by any treatment compared with control group (Supplementary Figure S1), histological analysis revealed that HISPRO significantly increased muscle fiber cross-sectional area (CSA) with a shift in fiber size distribution toward larger fibers (Figure 1E–G). Succinate dehydrogenase (SDH) staining further revealed a higher proportion of oxidative (SDH-positive) muscle fibers in the HISPRO-treated group than in the control group (Figure 1G), indicating a shift toward an oxidative muscle phenotype. Consistently, in vivo oxygen consumption rate was significantly enhanced by HISPRO compared with the control group (Figure 1H and Supplementary Figure S2). These findings indicate that HISPRO enhances muscle function and promotes an oxidative muscle phenotype, reflecting an overall improvement in muscle quality. Histidine alone modestly improved grip strength without significantly affecting rota-rod or treadmill performance compared with the control group, whereas proline alone showed minimal effects, indicating that the full improvement in motor coordination and endurance required the combined HISPRO treatment. Consistently, comparative analysis across histidine alone, proline alone, and HISPRO in skeletal muscle tissues showed that the combined treatment elicited the strongest induction of mitochondrial biogenesis-related genes (Supplementary Figure S3).

### 3.2. HISPRO Improved Mitochondrial Function and Oxidative Metabolism in Skeletal Muscle

**Figure 2:**
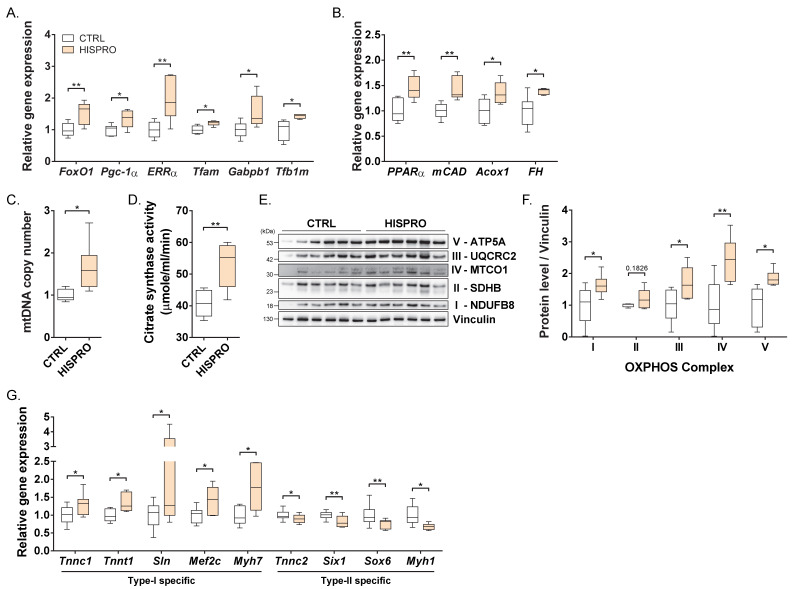
HISPRO promotes mitochondrial biogenesis and oxidative metabolism in skeletal muscle. (**A**) Expression of genes involved in mitochondrial biogenesis and maintenance in gastrocnemius muscle. Normalized to *Gapdh*. (**B**) Expression of fatty acid oxidation and oxidative metabolism-related genes in gastrocnemius. Normalized to *Gapdh*. (**C**) mtDNA copy number in gastrocnemius measured by D-loop quantification. (**D**) Citrate synthase activity in gastrocnemius. (**E**) Representative Western blot images of OXPHOS complex protein expression in gastrocnemius muscle. (**F**) Quantification of each complex protein. (**G**) Expression of muscle-type-specific genes in gastrocnemius. Normalized to *β-actin* or *Hprt*. *n* = 6 per group. Unpaired Student’s *t*-tests. * *p* < 0.05, ** *p* < 0.01.

To investigate the molecular basis underlying these functional improvements, we examined mitochondrial and metabolic gene expression in skeletal muscle. HISPRO treatment significantly upregulated genes associated with mitochondrial biogenesis (*Pgc-1α*, *ERRα*, *Tfam*, *Tfb1m*, *Gabpb1*), mitochondrial dynamics (*Mfn2*, *Drp1*), and metabolic regulation (*FoxO1*, *Sirt1*) compared with the control group (Figure 2A and Supplementary Figure S3). Consistently, genes involved in fatty acid oxidation and oxidative metabolism including *PPARα*, *mCAD*, *Acox1*, and *FH* were also significantly increased compared with the control group (Figure 2B). In line with these transcriptional changes, mtDNA copy number and citrate synthase activity were significantly elevated in the HISPRO-treated group compared with the control group (Figure 2C,D), indicating enhanced mitochondrial content and function. Moreover, protein levels of oxidative phosphorylation (OXPHOS) complexes were increased, with significant increases observed in complexes I, III, IV, and V compared with the control group (Figure 2E,F). In addition, compared with the control group, several genes associated with type I (slow-twitch) muscle fibers were upregulated whereas those of type II (fast-twitch) fibers were downregulated in the HISPRO treated gastrocnemius muscle (Figure 2G). These results suggest that HISPRO promotes mitochondrial biogenesis and drives a shift toward a more oxidative muscle phenotype.

### 3.3. HISPRO Enhanced SIRT1 Activity and AMPK Phosphorylation in Skeletal Muscle

**Figure 3:**
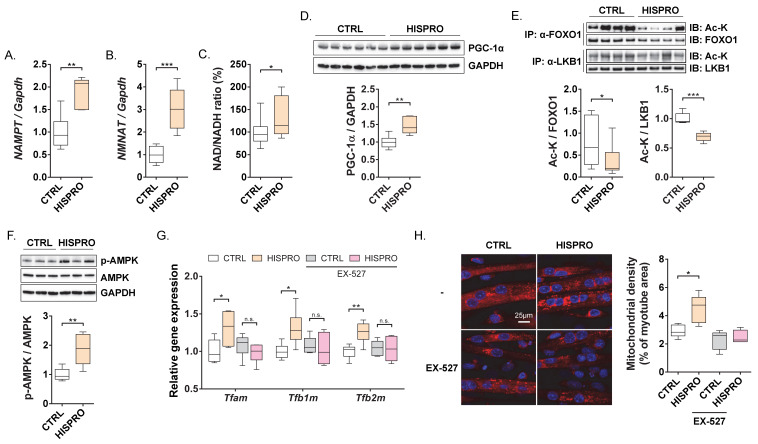
HISPRO activates SIRT1-dependent deacetylation and energy metabolism signaling. (**A**,**B**) Expression of genes involved in NAD^+^ synthesis in the gastrocnemius muscle. (**C**) NAD^+^/NADH ratio in gastrocnemius. (**D**) Representative Western blot images and quantified protein levels of PGC-1α in gastrocnemius. (**E**) Representative IP–Western blot images showing FOXO1 and LKB1 acetylation in gastrocnemius, and their quantification. (**F**) Representative Western blot images and quantified protein levels of p-AMPK and total AMPK in gastrocnemius. (**G**) Expression of genes involved in mitochondrial biogenesis and function in C2C12 myotubes in the presence of SIRT1 inhibitor EX-527. n.s., not significant. (**H**) Representative images of mitochondria stained with MitoTracker and quantification of mitochondrial density in C2C12 myotubes. Scale bar, 25 μm. *n* = 6 per group for (**A**–**F**), and *n* = 5 per group for (**G**,**H**). Unpaired Student’s *t*-tests. * *p* < 0.05, ** *p* < 0.01, *** *p* < 0.001.

Based on the observed transcriptional changes, we hypothesized that HISPRO activates the SIRT1 signaling axis, a key regulator of cellular energy metabolism. Consistent with this, HISPRO treatment significantly increased NAD^+^ synthesis-related genes including *NAMPT* and *NMNAT*, as well as the NAD^+^/NADH ratio, compared with the control group (Figure 3A–C), which is consistent with enhanced SIRT1 activity. HISPRO also increased PGC-1α protein levels and decreased acetylation of FOXO1 and LKB1 compared with the control group (Figure 3D,E). In addition, as LKB1 is an upstream kinase of AMPK [18], HISPRO treatment significantly increased AMPK phosphorylation compared with the control group (Figure 3F), indicating activation of the SIRT1–PGC-1α–AMPK signaling axis in skeletal muscle.

### 3.4. SIRT1 Inhibition Largely Suppressed HISPRO-Induced Mitochondrial Biogenesis but Did Not Fully Prevent OXPHOS Upregulation in C2C12 Myotubes

To determine whether the metabolic effects of HISPRO are dependent on SIRT1 activity, we evaluated its effects in C2C12 myotubes under SIRT1-inhibited conditions using EX-527. Treatment with 5 mM HISPRO significantly increased the expression of mitochondrial biogenesis-related genes including *Tfam*, *Tfb1m*, and *Tfb2m* compared with the control group. However, this effect was markedly attenuated by co-treatment with EX-527 (Figure 3G). Consistently, the HISPRO-induced increase in mitochondrial density was also reduced in the presence of EX-527 (Figure 3H), indicating that the mitochondrial biogenesis effects of HISPRO are largely dependent on SIRT1 activity. In contrast, OXPHOS complex proteins still showed a non-significant trend toward higher levels in HISPRO-treated cells compared with the control group even under SIRT1 inhibition, although these differences did not reach statistical significance (Supplementary Figure S4), suggesting that HISPRO’s effects on OXPHOS may involve additional mechanism(s) that are at least partly SIRT1-independent.

### 3.5. HISPRO Protected Against Dexamethasone-Induced Muscle Atrophy and Restored Mitochondrial Function

**Figure 4:**
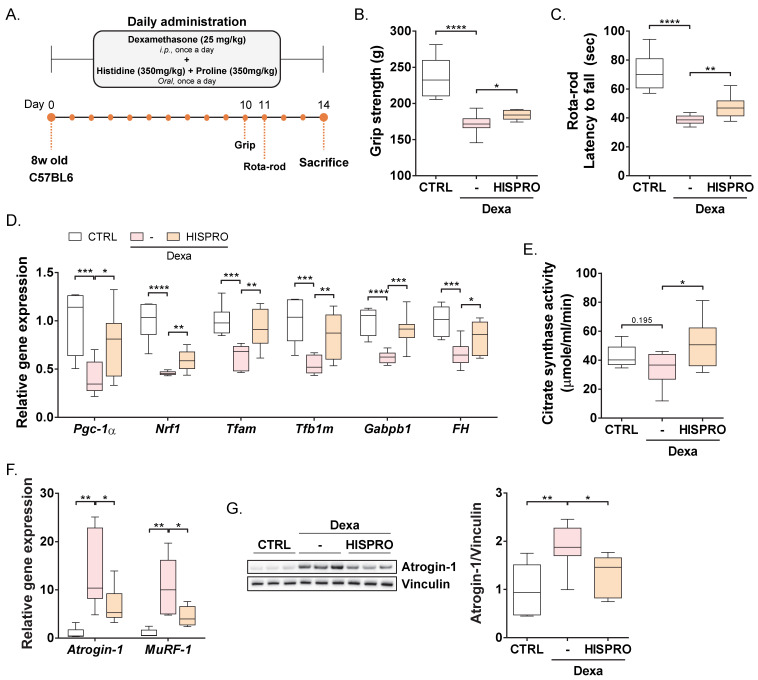
HISPRO protects against dexamethasone-induced muscle atrophy and improves mitochondrial function. (**A**) Experimental scheme. (**B**) Grip strength. (**C**) Endurance on rota-rod test. (**D**) Expression of genes involved in mitochondrial biogenesis and maintenance in the gastrocnemius muscle. (**E**) Citrate synthase activity in gastrocnemius muscle. (**F**) Atrophy-related gene expression in gastrocnemius muscle. Normalized to *Hprt*. (**G**) Atrogin-1 protein expression in gastrocnemius muscle. CTRL, *n* = 6; Dexa, *n* = 8; Dexa + HISPRO, *n* = 8. Unpaired Student’s *t*-tests. * *p* < 0.05, ** *p* < 0.01, *** *p* < 0.001, **** *p* < 0.0001.

Extending our findings to a muscle-wasting context, we further assessed the beneficial effects of HISPRO in a dexamethasone-induced muscle atrophy model (Figure 4A). Dexamethasone treatment markedly impaired muscle function, whereas HISPRO administration significantly improved muscle performance compared with the dexamethasone-treated group (Figure 4B,C). Consistent with our observations in normal mice, HISPRO restored the expression of genes involved in mitochondrial biogenesis and function that were suppressed by dexamethasone (Figure 4D). Moreover, citrate synthase activity was increased by HISPRO treatment compared with the dexamethasone-treated group (Figure 4E), indicating improved mitochondrial function. Notably, the expression of muscle atrophy-related genes including *Atrogin-1* and *MuRF1* was significantly downregulated by HISPRO treatment compared with the dexamethasone-treated group (Figure 4F), and Atrogin-1 protein level was consistently reduced (Figure 4G). Collectively, these results demonstrate that HISPRO mitigates dexamethasone-induced muscle wasting, at least in part, through restoration of mitochondrial function.

## 4. Discussion

Histidine-containing dipeptides and amino acid mixtures have been implicated in the regulation of skeletal muscle performance and mitochondrial metabolism, but the effects of a defined histidine–proline combination on oxidative muscle adaptation remain unclear. In particular, whether such supplementation engages the SIRT1–PGC-1α-AMPK axis has not been examined. In the present study, we found that HISPRO improves muscle function, increases oxidative fiber characteristics, and promotes mitochondrial biogenesis through activation of AMPK, SIRT1, and FOXO1/PGC-1α signaling, identifying HISPRO as a previously unrecognized amino acid combination that links nutrient availability to oxidative muscle remodeling. The increase in PGC-1α protein levels suggests a shift toward a more oxidative muscle phenotype, potentially reflecting fiber type switching toward oxidative muscle fibers. These findings indicate that HISPRO enhances muscle quality by improving both mitochondrial content and oxidative capacity in skeletal muscle.

To further understand the molecular mechanisms underlying HISPRO-mediated mitochondrial metabolic regulation, we examined the effects of SIRT1 inhibition. While SIRT1 inhibition markedly reduced HISPRO-induced mitochondrial biogenesis, it did not completely suppress the upregulation of OXPHOS protein levels. The observed partial SIRT1 dependence suggests that HISPRO may act through both SIRT1-dependent and additional SIRT1-independent pathways, although the precise mechanisms remain to be established. The SIRT1-dependent pathway promotes transcriptional activation of mitochondrial biogenesis via PGC-1α and related regulators [19]. In contrast, the preserved OXPHOS protein levels under SIRT1 inhibition suggest that HISPRO may also engage complementary SIRT1-independent mechanisms, potentially involving metabolic environment, redox signaling, or post-transcriptional and translational regulations.

As histidine and proline have distinct metabolic roles, their combined effects may complement each other to support cellular metabolism. Histidine-mediated enhancement of NAD^+^ metabolism likely promotes SIRT1 activation and downstream transcriptional regulations, whereas proline metabolism through the proline–P5C cycle may directly support mitochondrial electron transport and energy production [11,12,20]. Histidine supplementation may enhance NAD^+^ availability through one-carbon and NAD^+^ biosynthetic pathways [20], which would in turn favor SIRT1 activation and transcriptional programs of mitochondrial biogenesis [19]. In parallel, proline oxidation via the proline–P5C cycle directly feeds electrons into the respiratory chain and supports mitochondrial redox balance [12], thereby contributing to OXPHOS capacity independently of SIRT1. The persistence of elevated OXPHOS complexes under EX-527 treatment is compatible with such a SIRT1-independent, proline-driven contribution to the ETC function, but this interpretation remains speculative and will require direct testing in future studies.

From a physiological perspective, the ability of HISPRO to improve muscle function without significantly increasing muscle mass highlights its role in enhancing muscle quality rather than hypertrophy. This distinction is particularly important in aging and disease-associated muscle dysfunction, where improvements in metabolic efficiency and mitochondrial function are more relevant to functional outcomes than increases in muscle size alone [21,22,23]. Furthermore, the protective effects observed in the dexamethasone-induced muscle atrophy model indicate that HISPRO not only enhances baseline muscle performance but also confers resilience against catabolic stress, likely through preservation of mitochondrial integrity and suppression of atrophy-related pathways.

This study has several limitations. First, we examined young male mice fed a chow diet; therefore, species-specific differences should be considered when translating these findings to humans, and whether HISPRO exerts similar benefits in aged animals, females, or under metabolically stressed conditions remains to be determined. Second, although HISPRO consists of naturally occurring amino acids, long-term safety and toxicity following chronic administration at high supplemental doses were not fully evaluated. Third, although we observed clear activation of the SIRT1–PGC-1α–AMPK axis and partial SIRT1 dependence, we did not directly assess SIRT1 enzymatic activity or perform genetic loss-of-function experiments. In addition, although comparative in vivo analyses of histidine alone, proline alone, and HISPRO were performed, the precise molecular basis of the cooperative effect remains to be established. Finally, translation to humans will require clinical studies to establish efficacy and safety, as well as optimal dosing in target populations.

## 5. Conclusions

In conclusion, our study demonstrates that combined histidine and proline supplementation enhances skeletal muscle function by promoting mitochondrial biogenesis and oxidative remodeling, in part via activation of the SIRT1–PGC-1α-AMPK signaling axis, while also engaging complementary SIRT1-independent mechanisms to support mitochondrial function. These findings identify HISPRO as a novel nutritional strategy, positioning HISPRO as a promising nutritional intervention for conditions characterized by mitochondrial dysfunction and muscle wasting.

## Data Availability

The original contributions presented in this study are included in the article/Supplementary Material. Further inquiries can be directed to the corresponding author.

## Supplementary Materials

The following supporting information can be downloaded at https://www.mdpi.com/article/10.3390/cells15100887/s1. Figure S1: Body and muscle weights; Figure S2: In vivo oxygen consumption (VO_2_) rate over time; Figure S3: Expression of genes involved in mitochondrial biogenesis and metabolism in skeletal muscle tissues across experimental groups; Figure S4: OXPHOS complex protein expression in C2C12 myotubes in the presence of the SIRT1 inhibitor EX-527; Figure S5: Body weights under dexamethasone-induced muscle atrophy conditions in C57BL/6 mice; Table S1: Primer sequence.
