# Supplementary material for: Combined Histidine and Proline Supplementation (HISPRO) Enhances Oxidative and Mitochondrial Function in Skeletal Muscle Through SIRT1-Associated Signaling
Source: Cells. 2026 May 13;15(10):887. doi: 10.3390/cells15100887 (PMC13204685; doi:10.3390/cells15100887)
Supplement: Supplementary file 1 [file cells-15-00887-s001.zip › cells-4261003-supplementary.pdf]

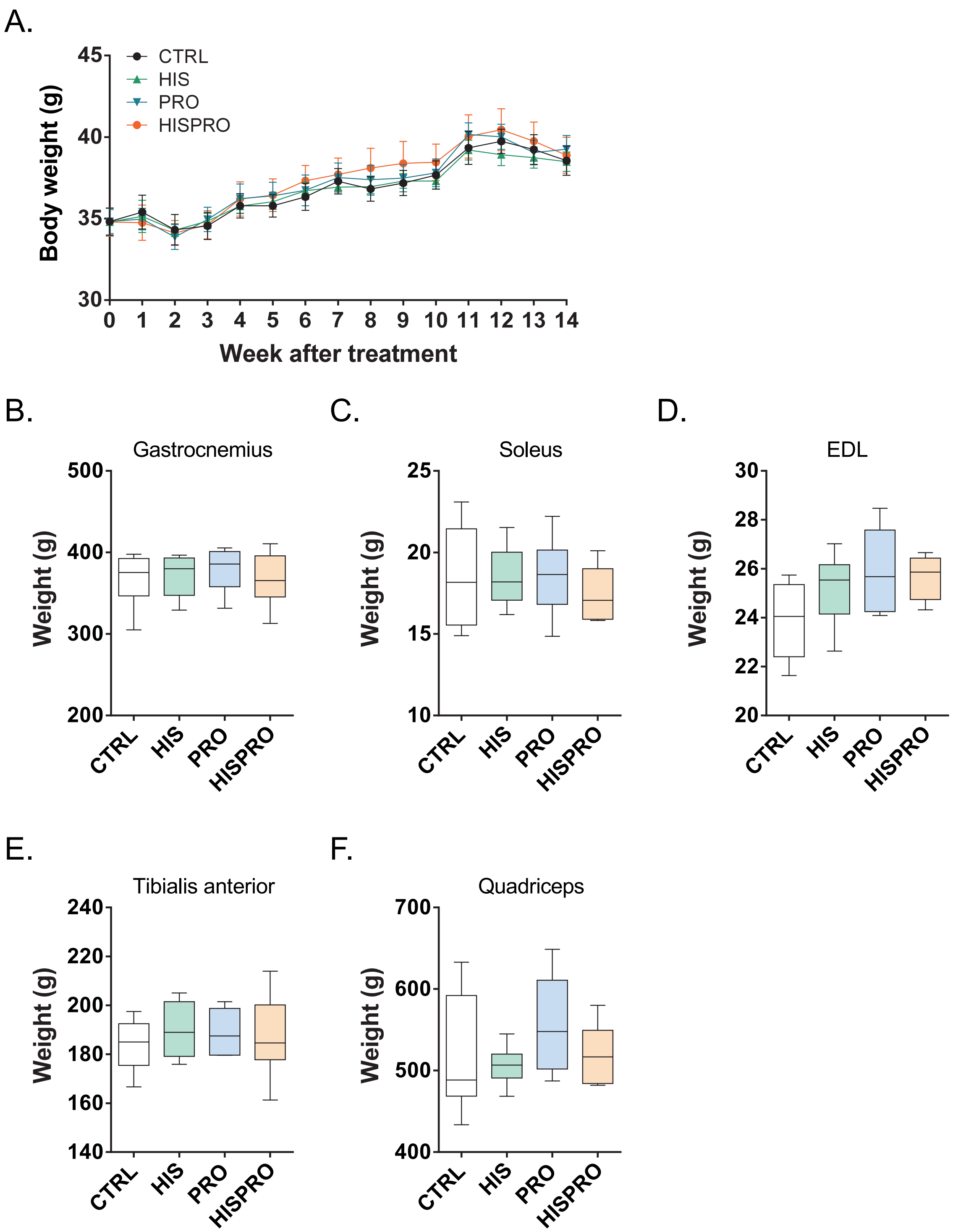

Supplementary Figure S1. Body and muscle weights under physiological conditions in ICR mice. (A) Changes in body weight during the treatment period. Data are presented as mean ± SEM. (B-F) Weights of gastrocnemius, soleus, EDL, Tibialis anterior, and quadriceps muscles. Data are presented as box-and-whisker plots (median, IQR, and min-to-max whiskers).

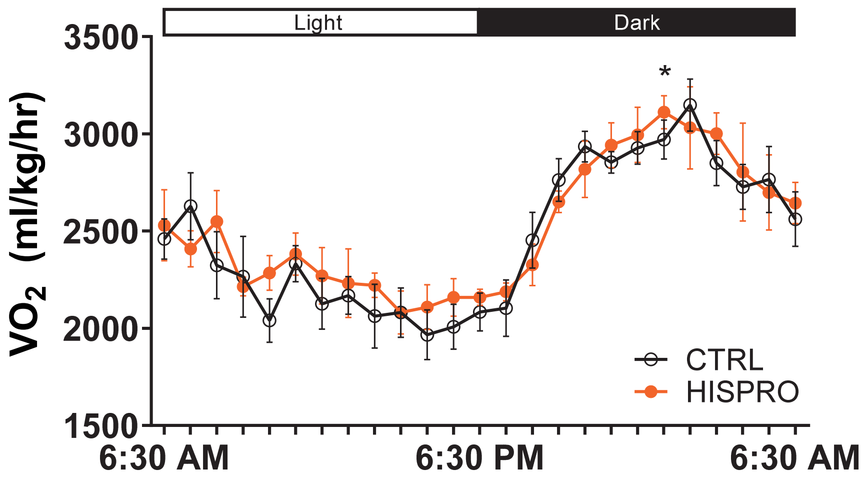

Supplementary Figure S2. In vivo oxygen consumption (VO_2) rate over time. Data are presented as mean ± SEM. Unpaired Student's t-test. *$p < 0.05$.

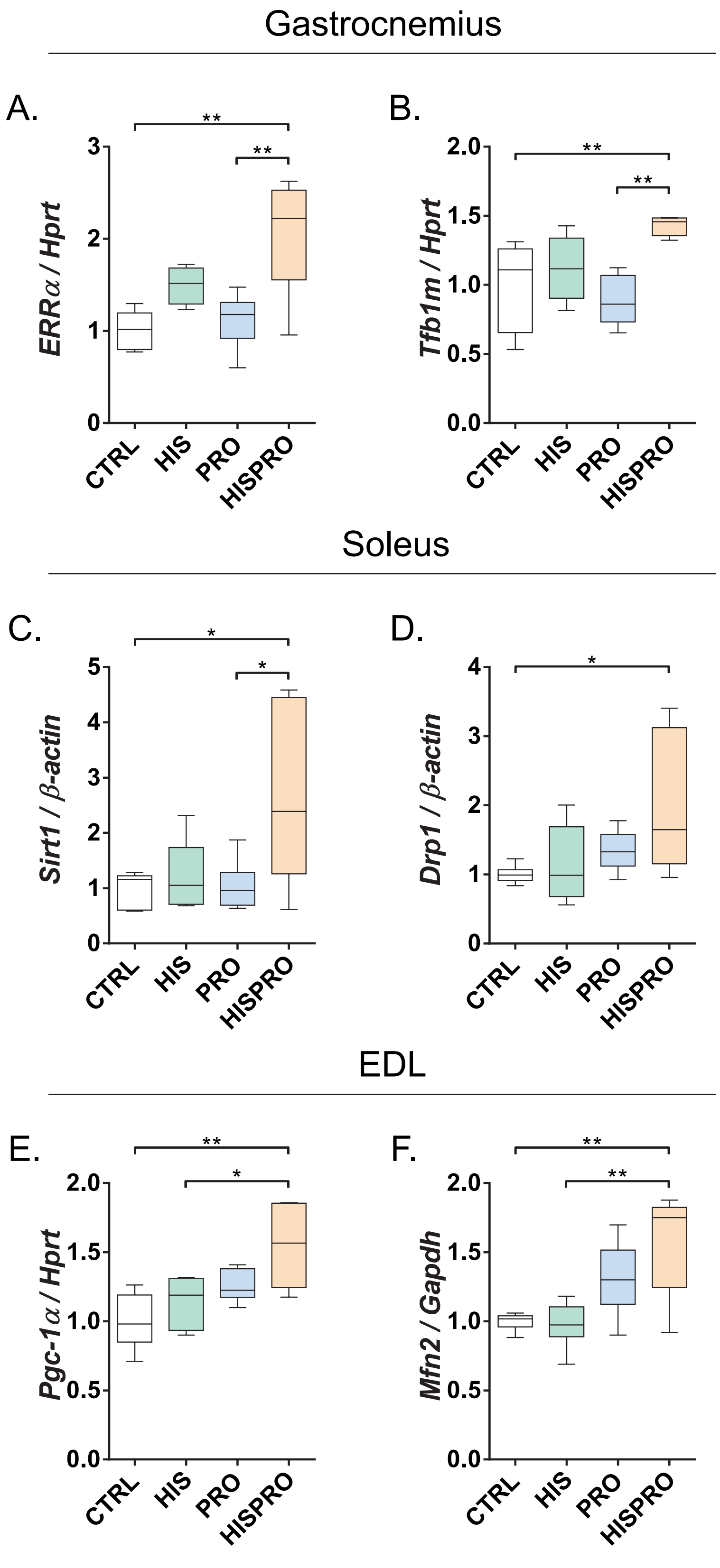

Supplementary Figure S3. Expression of genes involved in mitochondrial biogenesis and metabolism in skeletal muscle tissues across experimental groups. (A, B) Gene expressions in gastrocnemius. (C, D) Gene expressions in soleus. (E, F) Gene expressions in EDL. Data are presented as box-and-whisker plots (median, IQR, and min-to-max whiskers). One-way ANOVA with Sidak's multiple comparison test. $*p < 0.05$, $**p < 0.01$.

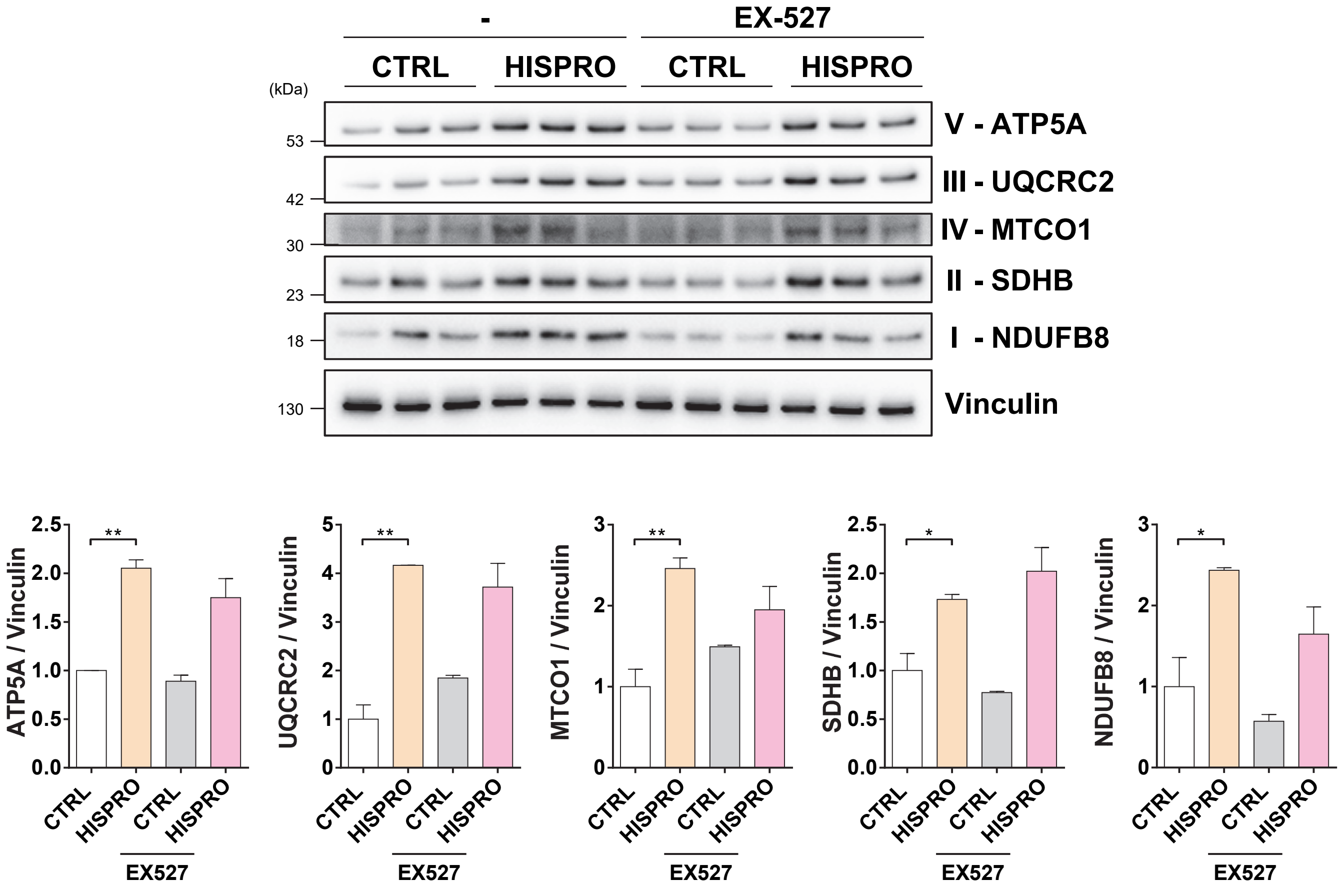

Supplementary Figure S4. OXPHOS complex protein expression in C2C12 myotubes in the presence of the SIRT1 inhibitor EX-527. Data are presented as mean ± SEM. Unpaired Student’s t-test. *p < 0.05, **p < 0.01.

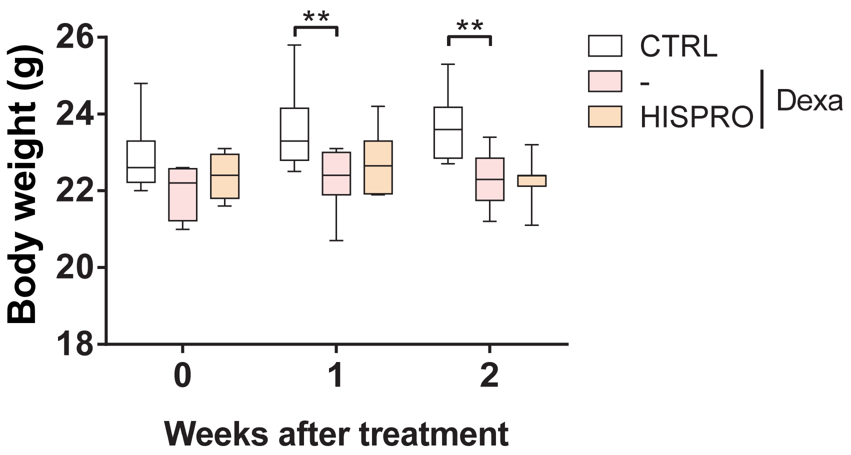

Supplementary Figure S5. Body weight changes under dexamethasone-induced muscle atrophy conditions in C57BL/6 mice. Data are presented as box-and-whisker plots (median, IQR, and min-to-max whiskers). Two-way ANOVA with Sidak's multiple comparison test. **p < 0.01.

Supplementary Table S1. Primer sequence.

Gene name	Forward primer (5' to 3')	Reverse primer (5' to 3')
Esrra	CAGGAGGCAGACACTGAT	CGGATTAAGCAGCAGCAA
Tfb1m	GGCTGAGAGACTTGTAGCCACT	AGGTGCACCACTCCTACATCAA
Tfb2m	TTTGGCAAGTGGCCTGTGAC	ACTGATTCCCCGTGCTTTGACT
Sirt1	GTTGACCGATGGACTCCTCAC	GAGCTGGCGTGTGACGTTC
Drp1	AGGAGAAGAGGAAGCAAGCG	TAGGCTTTCCAGCACTGAGC
Ppargc1a	AAGGACTCTGAGAACACTTG	CAACTGACCCAAACACTTTAC
Mfn2	GCTTGGACAGGTGGAGTCAA	CAGGGACATCTCGCCAGTTT
Foxo1	CCTTTCCTCCTCCCTCTG	TGCCTCTACTGAATGATTACA
Tfam	CGGACCTCTAAGATCTAACTAC	CTACCTTTCCCATTCCCTTC
Nrf1	CCTCAGCCTCCATCTTCT	GACCTTACAACCAAGCAACT
Gabpb1	AGTGGCCACAGAGGAAGTG	TGATGACTTGCTGACCCCCT
Ppara	ACTTGCCTCACTACTGTCCTT	TGCTGGTATCGGCTCAATA
Acadm	TAGACGAAGCCACGAAGTA	GAGCCTAGCGAGTTCAAC
Acox1	ACACTAACATATCAACAAGAGGAG	CATTGCCAGGAAGACCAG
Tnnc1	GATGGTTCGGTGCATGAAGGAC	CTTCCGTAATGGTCTCACCTGTG
Tnnt1	GAGCAGAGGATGACGCCAAGAA	TTCATCTCCCGACCAGTCTGTC
Sln	ACTGAGGTCCTTGGTAGCCT	CATGGCCCCTCAGTATTGGT
Mef2c	GTGGTTTCCGTAGCAACTCCTAC	GGCAGTGTTGAAGCCAGACAGA
Myh7	TTGCTACCCTCAGGTAGGAGT	CCTTTCTCGGAGCCACCTTG
Tnnc2	AGCGAAGAGGAACTGGCTGAGT	CGATCTCCTCTTCTGTCACATGC
Six1	AGGTCAGCAACTGGTTTAAGAACC	GAGTTGATTCTGCTTGTTGGAGG
Sox6	GCATAAGTGACCGTTTTGGCAGG	GGCATCTTTGCTCCAGGTGACA
β-actin	GGGAAGGTGACAGCATTG	ATGAAGTATTAAGGCGGAAGATT
Hprt	AAATGTCAGTTGCTGCGTCC	TCTACCAGAGGGTAGGCTGG
Myh1	GCATCCCTAAAGGCAGGCTC	CAAACACCGATGACTTGGCG
Nampt	AAGAGACTGCTGGCATAGGG	TTAGAGCAATTCCCGCCACA
Nmnat1	ATTGCTGTGTGGGGCAGATT	CCACGATTTGCGTGATGTCC
D-Loop	AAGGACATATCTGTGTTATCT	TTCACGGAGGATGGTAGATTA
18s rRNA	CAGTAAGTGCGGGTCATAA	CCATCCAATCGGTAGTAGC
